# Influence of 30 and 60 Min of Hypobaric Hypoxia in Simulated Altitude of 15,000 ft on Human Proteome Profile

**DOI:** 10.3390/ijms23073909

**Published:** 2022-03-31

**Authors:** Jan Schmitz, Lydia J. Kolaparambil Varghese, Felix Liebold, Moritz Meyer, Lukas Nerlich, Clement Starck, Seamus Thierry, Stefanie Jansen, Jochen Hinkelbein

**Affiliations:** 1 Department of Anaesthesiology and Intensive Care Medicine, Faculty of Medicine and University Hospital Cologne, University of Cologne, 50937 Cologne, Germany; felix.liebold@t-online.de (F.L.); jochen.hinkelbein@uk-koeln.de (J.H.); 2 German Society of Aerospace Medicine (DGLRM), 80331 Munich, Germany; lukas.nerlich@hotmail.de; 3 Space Medicine Group, European Society of Aerospace Medicine (ESAM), 51149 Cologne, Germany; lydiaj2397@gmail.com; 4 Department of Sleep and Human Factors Research, German Aerospace Center, Institute of Aerospace Medicine, 51147 Cologne, Germany; 5 Faculty of Medicine and Surgery, Università degli Studi di Perugia (Terni), 01500 Perugia, Italy; 6 Department of Otorhinolaryngology, Faculty of Medicine and University Hospital Essen, University of Essen, 45147 Essen, Germany; moritz.meyer@uk-essen.de; 7 Anesthesiology and Intensive Care Department, University Hospital of Brest, 29200 Brest, France; clementstarck@gmail.com; 8 Anesthesiology Department, South Brittany General Hospital, 56322 Lorient, France; seam.thi@gmail.com; 9 Head and Neck Surgery, Department of Otorhinolaryngology, Medical Faculty, University of Cologne, 50937 Cologne, Germany; stefanie.jansen@uk-koeln.de

**Keywords:** hypobaric hypoxia, proteomics, pressure, protein expression, plasma

## Abstract

The human body reacts to hypobaric hypoxia, e.g., during a stay at high altitude, with several mechanisms of adaption. Even short-time exposition to hypobaric hypoxia leads to complex adaptions. Proteomics facilitates the possibility to detect changes in metabolism due to changes in proteins. The present study aims to identify time-dependent changes in protein expression due to hypobaric hypoxia for 30 and 60 min at a simulated altitude of 15,000 ft. N = 80 male subjects were randomized and assigned into four different groups: 40 subjects to ground control for 30 (GC30) and 60 min (GC60) and 40 subjects to 15,000 ft for 30 (HH30) and 60 min (HH60). Subjects in HH30 and HH60 were exposed to hypobaric hypoxia in a pressure chamber (total pressure: 572 hPa) equivalent to 15,000 ft for 30 vs. 60 min, respectively. Drawn blood was centrifuged and plasma frozen (−80 °C) until proteomic analysis. After separation of high abundant proteins, protein expression was analyzed by 2-DIGE and MALDI-TOF. To visualize the connected signaling cascade, a bio-informatical network analysis was performed. The present study was approved by the ethical committee of the University of Cologne, Germany. The study registry number is NCT03823677. In comparing HH30 to GC30, a total of seven protein spots had a doubled expression, and 22 spots had decreased gene expression. In a comparison of HH60 to GC60, a total of 27 protein spots were significantly higher expressed. HH60, as compared to GC30, revealed that a total of 37 spots had doubled expression. Vice versa, 12 spots were detected, which were higher expressed in GC30 vs. HH60. In comparison to GC, HH60 had distinct differences in the number of differential protein spots (noticeably more proteins due to longer exposure to hypoxia). There are indicators that changes in proteins are dependent on the length of hypobaric hypoxia. Some proteins associated with hemostasis were differentially expressed in the 60 min comparison.

## 1. Introduction

With an increase in altitude, atmospheric pressure and the partial pressure of oxygen (O_2_) decrease [1], organisms at higher altitudes must adapt to the stress of limited oxygen availability [2]. This condition is termed hypobaric hypoxia (HH), which stresses biological systems because of the non-availability of the oxygen supply for mitochondrial metabolism [1]. The reduced partial pressure of oxygen (paO_2_) can trigger the onset of adaptive responses [3,4], resulting in complex changes in the expression of genes, even after a short-term exposure to HH [5,6]. This causes an alteration in the expression and number of genes, including stress-related genes and corresponding proteins that are necessary to maintain homeostasis [1,4].

Changes in specific gene expression levels as well as the protein links are excellent indicators for the adaption of metabolic pathways in response to hypoxic stress [1,7]. However, little is known about protein expression during and after short-term and moderate hypoxia. For research of hypoxia-induced alterations in protein expression, various technologies have been available for several years. Those technologies are summarized under the term “proteomics” [8], but the analysis of complex contexts failed for many years due to a large amount of generated data. Within the last few years, computer-based systems have been introduced showing new possibilities to systematically analyze signaling pathways [9]. For example, two-dimensional gel electrophoresis (2-DIGE) coupled with mass spectrometry (MS) for the identification of proteins is a powerful approach to understanding global protein dynamics in response to different stimuli [10]. The combination with bio-informatic network analyses is an integral technology to identify the change of innumerable proteins at a defined time [11].

Currently, only very few investigators have applied these technologies to the study of humans at (simulated) altitude and only under a limited number of hypoxic conditions and durations, and with usually small sample sizes [1]. Hence, previous data suggested that hypobaric hypoxia during airline travel (2300 m or 8000 ft) induces several (patho-) physiological reactions in the human body [12,13,14,15] as well as an association in signaling pathways, for example, in immune response, protein metabolism and hemostasis [6].

The presented study aimed to measure the influence of hypobaric hypoxia at a simulated altitude of 15,000 ft on protein expression in the serum of healthy volunteers. To our knowledge, this is the first study designed to examine the effect of hypobaric hypoxia at 15,000 ft in a pressure chamber on the human proteome.

## 2. Results

### 2.1. Comparison 1: Hypobaric Hypoxia 30 min (HH30) versus Ground Control 30 min (GC30)

**Figure 1:**
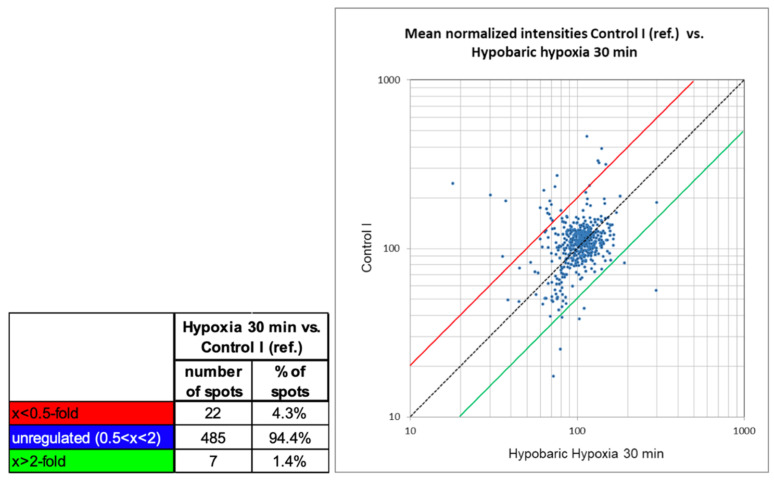
Summary of quantitative changes of comparison 1: HH30 versus GC30.

An overlay image of the gels hypobaric hypoxia 30 min and GC30 was generated with Delta 2D. A 2-fold change was set as the regulation threshold the quantitative comparison. Figure 1 summarizes of the quantitative differences as found with the image analysis. The number of un-changed protein spots between the two samples (found to be different below factor 2 and above 0.5) was high in 94.4% of all protein spots. A total of 7 protein spots were found to be >2-fold higher expressed in the sample HH30, and 22 protein spots were found to be >2-fold higher expressed in the sample GC30. As seen in the scatter plot (Figure 1), the intensity of the protein spots found differentially expressed varies from very faint spots (localized in the lower part of the graph) to higher abundant protein spots (localized in the upper part of the graph).

**Figure 2:**
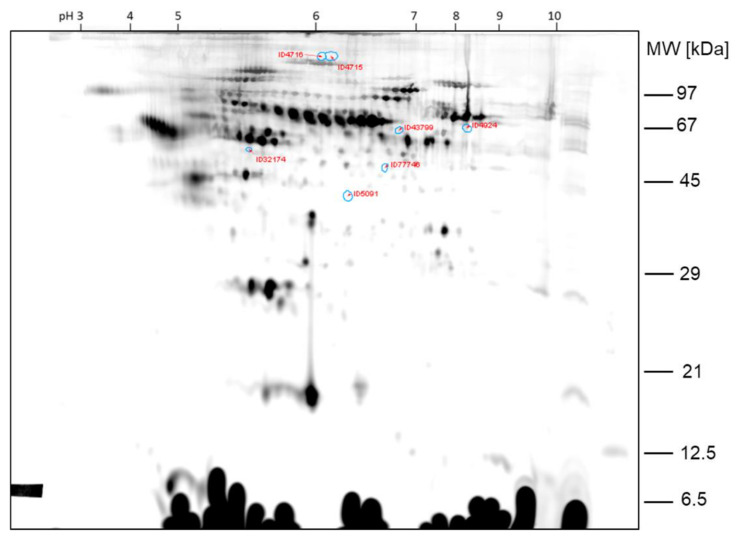
Comparison 1: Spots found >2-fold higher expressed in sample HH30.

**Figure 3:**
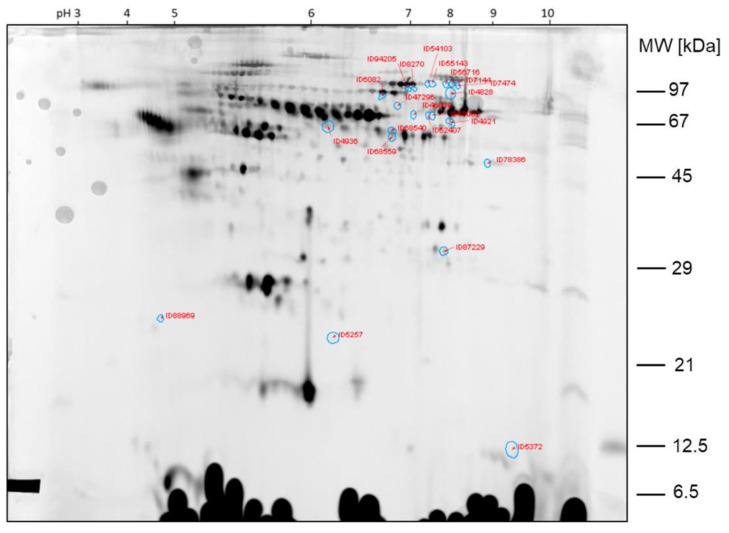
Comparison 1: Spots found >2-fold higher expressed in GC30.

The differentially expressed protein spots between the two samples of comparison 1 are shown in the 2D gel image in Figure 2 and Figure 3. Spot ratios >1 indicate a higher spot intensity in sample HH30; spot ratios <1 indicate a higher spot intensity in sample GC30.

The spots ID87229, ID68540 and ID68559, which are >2-fold higher expressed in sample GC30 than in sample HH30, were already found by visual inspection of the gel. The other protein spots found differentially expressed by image analysis with Delta 2D were less obvious from the visual inspection of the 2D DIGE gels.

### 2.2. Bio-Informatic Network Analysis (Data Query: 12 May 2020)

**Figure 4:**
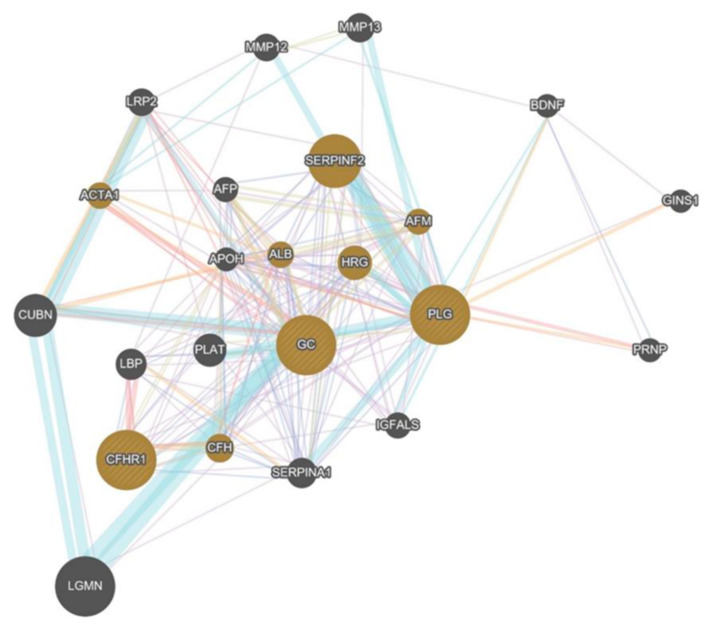
Bio-Informatical Analysis of HH30 vs. GC30.

Three differentially expressed proteins were linked to GeneMania, resulting in 282 connections. Mostly physical interactions (67.64%), co-expression (13.50%), forecast (6.35%), co-localization (6.17%), metabolic pathways (4.35%), gen-interaction (1.40%) and shared-protein-domains (0.59%) were detected. Lowest FDR (0.0000000000505) was analyzed for microparticles of blood (GC; PLG; CFHR1) but allows no precise statement about pathways (Figure 4).

### 2.3. Comparison 2: Hypobaric Hypoxia 60 min (HH60) versus Ground Control 60 min (GC60)

An overlay image of the gels HH60 and GC60 was generated with Delta 2D. The protein pattern of GC60 is visualized in orange; the protein pattern of HH60 is shown in blue. Protein spots expressed equally strong in both samples, resulting in a complete overlay seen as blackish–brown spots. The protein spots, already found differentially expressed by visual inspection of the DIGE images of the replicate gels, are retrieved in the overlay image of the two fusion gels, as indicated by the clearly orange and blue color of the respective protein spots.

**Figure 5:**
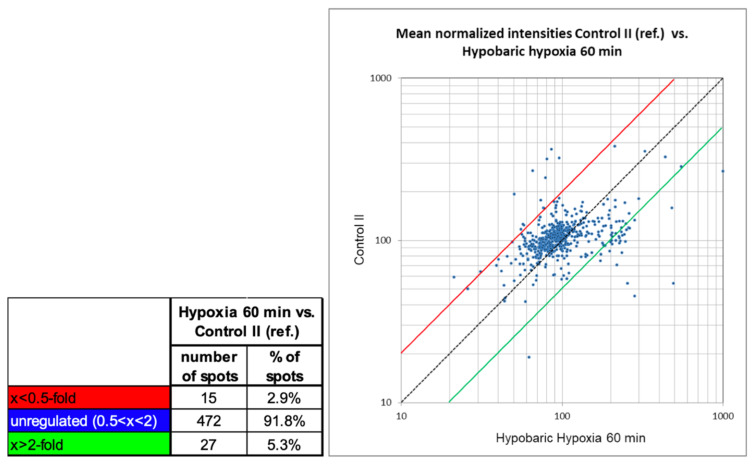
Summary of quantitative changes of comparison 2: HH60 versus GC60.

A 2-fold change was set as the threshold of regulation for the quantitative comparison. Figure 5 shows a summary of the quantitative differences as found with the image analysis. The number of un-changed protein spots between the two samples (found to be different below factor 2 and above 0.5) was high in 91.8% of all protein spots. A total of 27 protein spots were found to be >2-fold higher expressed in the sample HH60, and 15 protein spots were found to be >2-fold higher expressed in the sample GC60. As seen in the scatter plot (Figure 5), the intensity of the protein spots found differentially expressed varies from very faint spots (localized in the lower part of the graph) to higher abundant protein spots (localized in the upper part of the graph).

**Figure 6:**
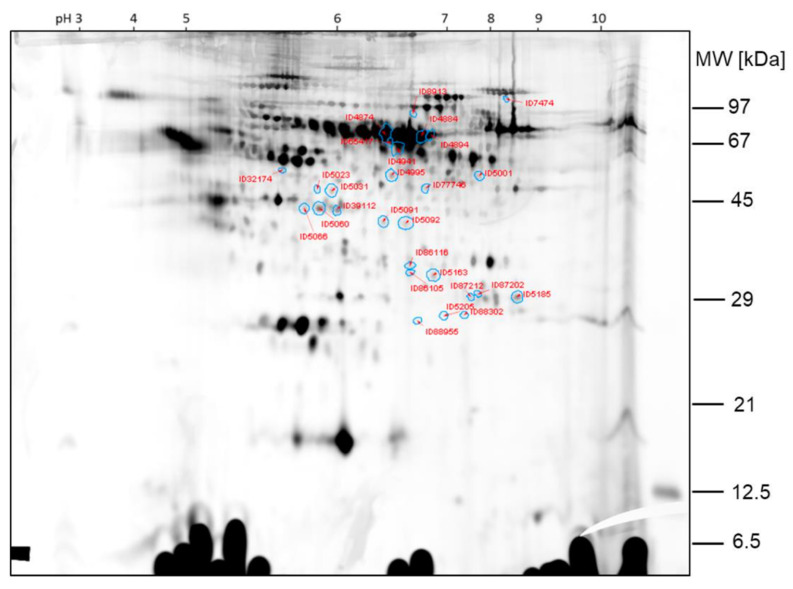
Comparison 2: Spots found >2-fold higher expressed in sample HH60.

**Figure 7:**
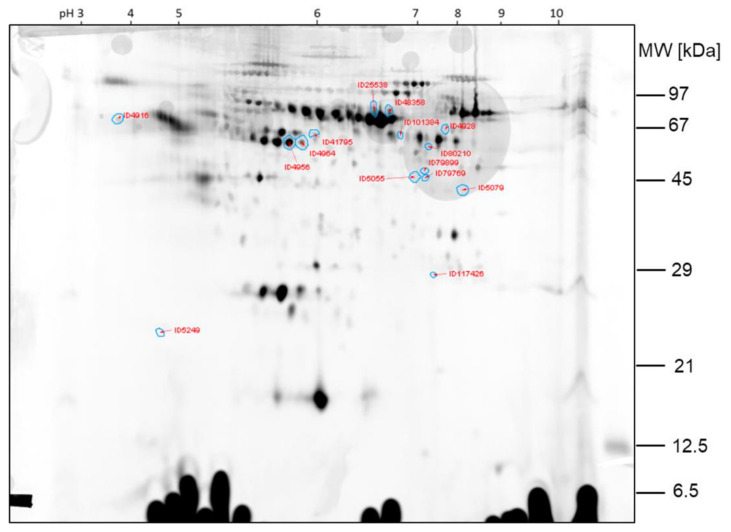
Comparison 2: Spots found >2-fold higher expressed in GC60.

The protein spots that were found differentially expressed between the two samples of comparison 2 are shown in the 2D gel image in Figure 6. Spot ratios >1 indicate a higher spot intensity in sample hypobaric hypoxia 60 min; spot ratios <1 indicate a higher spot intensity in GC60 (Figure 7).

The spots ID5066, ID5060, ID5185 and ID39112, which are >2-fold higher expressed in sample HH60 than in sample GC60, were already found by visual inspection of the gel. The other protein spots found differentially expressed by image analysis with Delta 2D were less obvious from the visual inspection of the 2D DIGE gels.

### 2.4. Bio-Informatic Network Analysis (Data Query: 12 May 2020)

**Figure 8:**
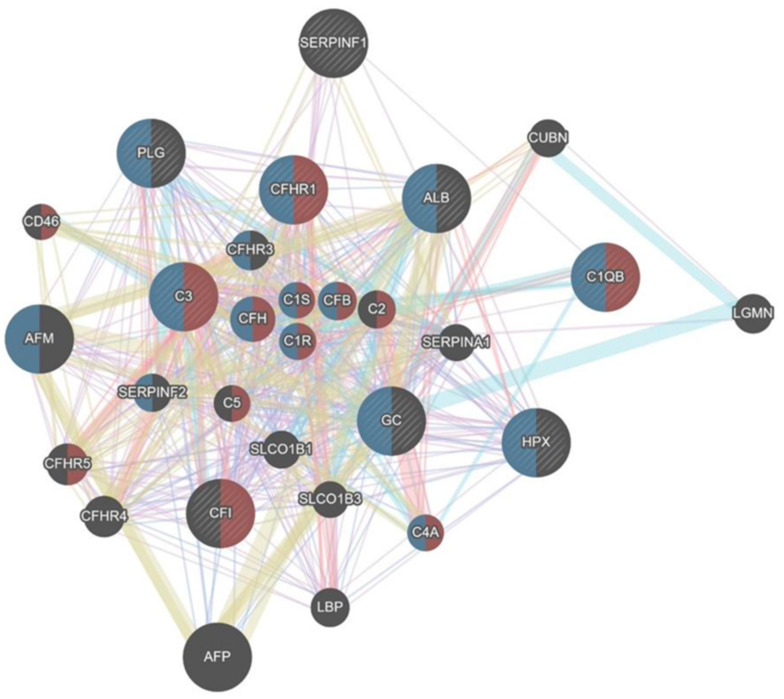
Bio-Informatical Network Analysis HH 60 vs. GC60.

In this comparison, a total of nine differential expressed proteins were linked to 20 GeneMania-related proteins, resulting in 735 connections. Most common links were co-expression (41.14%), shared-protein-domain (22.92%), co-localization (13.78%), physical interactions (13.03%) and signaling pathways (9.13%) (Figure 8). Metabolic pathways that seemed to be of relevance were the following:

Complement activating (FDR 4.78 × 10^−24^) (C3; CFHR1; C1QB; CFI)
Microparticle (Blood) (FDR 3.45 × 10^−23^) (C3; CFHR1; C1QB; HPX; GC; PLG)
Protein activating (FDR 2.82 × 10^−22^) (C3; CFHR1; C1QB; CFI)
Immune Response (FDR 4.96 × 10^−19^) (C3; CFHR1; C1QB; CFI)

### 2.5. Comparison 3: Hypobaric Hypoxia 60 min (HH60) versus Hypobaric Hypoxia 30 min (HH30)

An overlay image of the gels hypobaric hypoxia 60 min and hypobaric hypoxia 30 min was generated with Delta 2D. The protein spots already found differentially expressed by visual inspection of the DIGE images of the replicate gels are retrieved in the overlay image of the two fusion gels, as indicated by the clearly orange and blue color of the respected protein spots.

**Figure 9:**
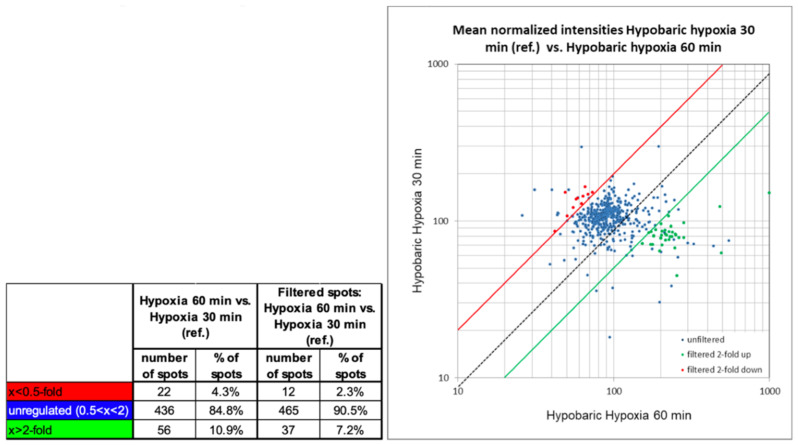
Summary of quantitative changes of comparison 3: HH60 versus HH30. Spot intensities prior and after filtering (for details, see text).

A 2-fold change was set as the threshold of regulation for the quantitative comparison. Figure 9 shows a summary of the quantitative differences as found with the image analysis.

The number of un-changed protein spots between the two samples (found to be different below factor 2 and above 0.5) was relatively high in 84.8% of all protein spots. A total of 56 protein spots were found to be >2-fold higher expressed in the sample hypobaric hypoxia 60 min, and 22 protein spots were found to be >2-fold higher expressed in the sample hypobaric hypoxia 30 min. These numbers of changed protein spots were relatively high. Careful evaluation of these particular spots revealed that for some of them the intensity values in the control samples (control I and/or control II) were also found changed (potentially false positive hits). Therefore, the protein spots found >2-fold higher expressed in the sample hypobaric hypoxia 60 min were filtered to eliminate any spots that additionally showed >2-fold higher intensity values in the control samples compared to hypobaric hypoxia 30 min. Furthermore, protein spots found >2-fold higher expressed in the sample hypobaric hypoxia 30 min were filtered to eliminate any spots that additionally showed >2-fold higher intensity values in the control samples compared to hypobaric hypoxia 60 min. As a result, 37 protein spots were found to be >2-fold higher expressed in the sample hypobaric hypoxia 60 min, and 12 protein spots were found to be >2-fold higher expressed in the sample hypobaric hypoxia 30 min after filtering.

As seen in the scatter plot (Figure 9), the intensity of the protein spots found differentially expressed varies from very faint spots (localized in the lower part of the graph) to higher abundant protein spots (localized in the upper part of the graph). The protein spots, after filtering to eliminate potential false positive hits, are depicted as red data points (>2-fold higher expressed in sample hypobaric hypoxia 30 min) and as green data points (>2-fold higher expressed in sample hypobaric hypoxia 60 min).

**Figure 10:**
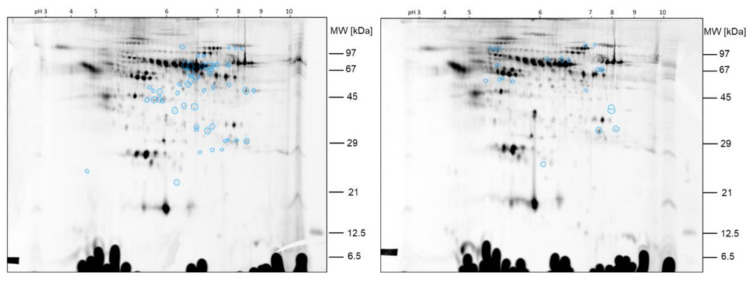
Comparison 3: Spots found >2-fold higher expressed in sample hypobaric hypoxia 60 min (**left**) versus >2-fold higher expressed in sample hypobaric hypoxia 30 min (**right**).

The protein spots that were found differentially expressed between the two samples of comparison 3 prior filtering for potential false positive hits are shown in the 2D gel image in Figure 10.

**Figure 11:**
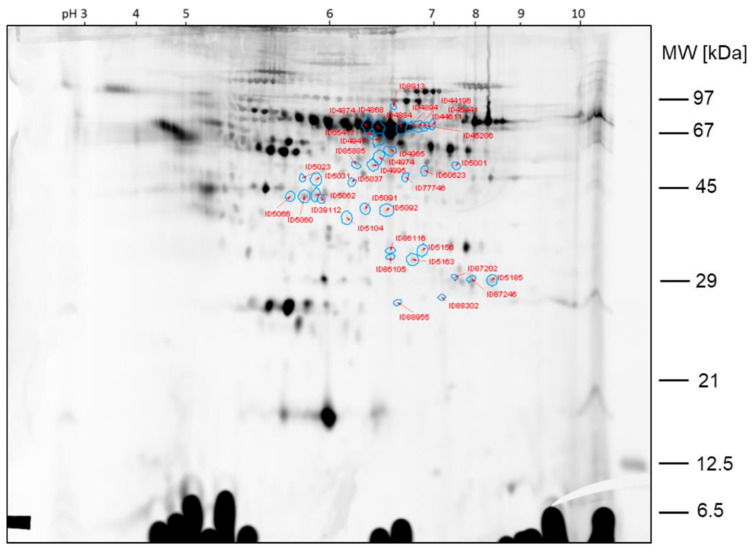
Comparison 3: Spots found >2-fold higher expressed in sample hypobaric hypoxia 60 min. Spots after filtering.

In Figure 11, the protein spots that were found differentially expressed between the two samples of comparison 3 after filtering are shown. Spot ratios >1 indicate a higher spot intensity in sample hypobaric hypoxia 60 min; spot ratios <1 indicate a higher spot intensity in sample hypobaric hypoxia 30 min.

The spots ID5066, ID5060, ID39112 and ID5185, >2-fold higher expressed in sample hypobaric hypoxia 60 min than in sample hypobaric hypoxia 30 min, were already found by visual inspection of the gel and already found by image analysis of comparison 2. Additionally, the spots ID60623 and ID87246, which are >2-fold higher expressed in sample hypobaric hypoxia 60 min than in sample hypobaric hypoxia 30 min, were also found by visual inspection and could be confirmed with image analysis. The other protein spots found differentially expressed by image analysis with Delta 2D were less obvious from the visual inspection of the 2D DIGE gels.

### 2.6. Bio-Informatical Network Analysis (Data Query: 12 May 2020)

**Figure 12:**
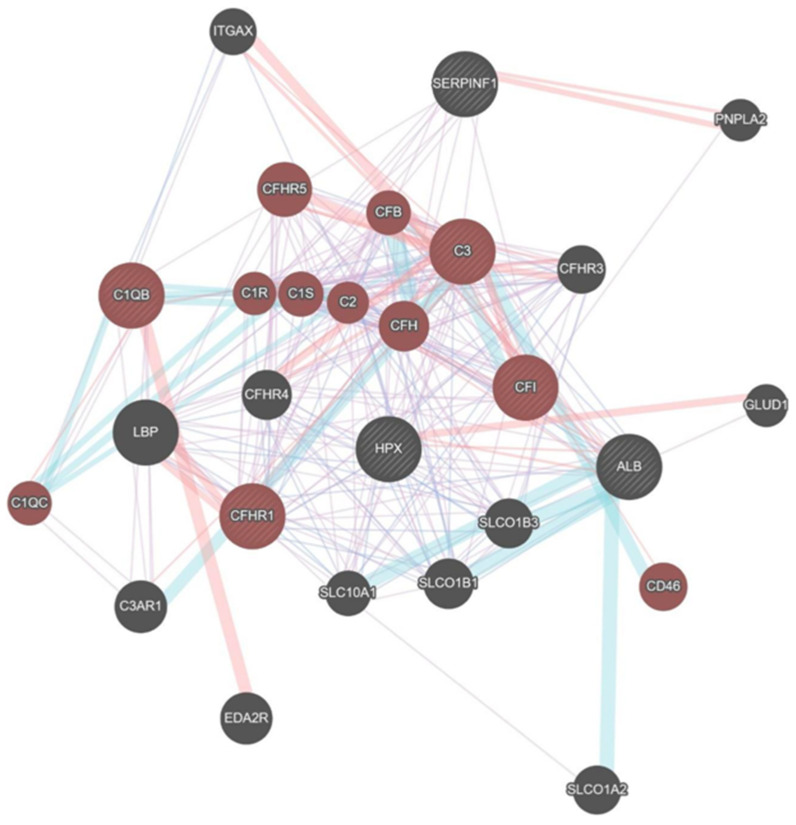
Bio-Informatical Network Analysis HH 60 vs. HH30.

Most common interactions were: Co-expression (41.74%), physical interaction (26.29%), pathways (23.47%) and co-localizations (8.50%). The differential expressed proteins were linked to a total of 357 connections. Changes in the complement system (FDR: 7.74 × 10^−22^), activation of proteins (FDR: 4.34 × 10^−20^) and immune response (FDR: 3.54 × 10^−17^) were shown to be associated most relevantly with hypobaric hypoxia. C3, CFHR1, C1QB and CFI were involved in three different pathways (Figure 12).

### 2.7. Summary of the Comparisons

**Figure 13:**
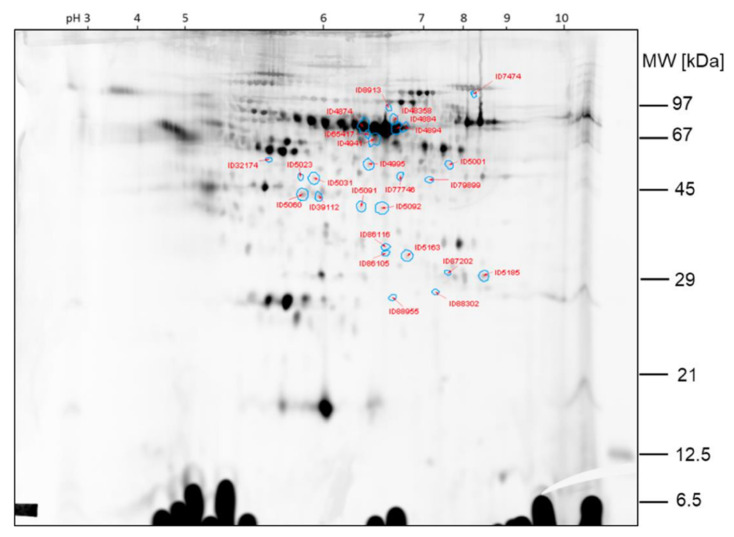
Spots found changed in more than one comparison.

**Figure 14:**
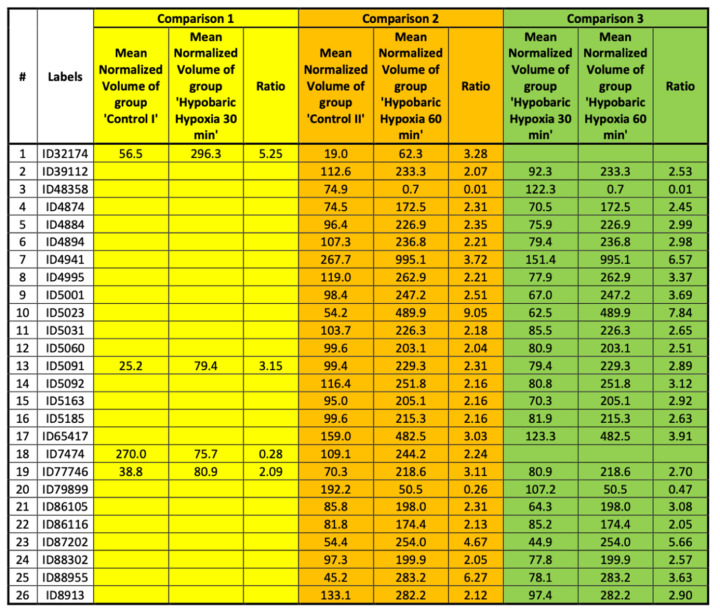
Spots found changed in more than one comparison.

The number of protein spots found differentially present in the two samples of comparison was slightly higher when comparing the sample hypobaric hypoxia 60 min to its respective control sample (comparison 2), as well as comparing the sample hypobaric hypoxia 30 min to its respective control (comparison 1). Protein spots that were found differentially expressed in more than one comparison are summarized in Figure 13 and Figure 14.

In total, 26 spots were found to be differentially expressed in the two compared samples in more than one comparison. Thus, an increased probability that the respective differences are originated from differences in the sample material rather than technical variability caused by sample handling during 2D protein separation can be assumed.

In principle, since only one replicate gel was produced per sample, it is not entirely clear if the found differences are all caused by differences in the sample material or if they are originated from technical variability. Additionally, the preparation of the samples performing the depletion of high-abundant proteins is probably not strictly reproducible. Therefore, it cannot be excluded that some differences between the gels may have been caused by differences in the protein depletion procedure.

## 3. Discussion

To analyze the effect of hypobaric hypoxia on humans, serum samples donated by 19–20 independent donors were pooled per experimental group. Serum taken after exposition to hypobaric hypoxia for 30 min and 60 min (15,000 ft) and from two control groups (no hypoxia) were analyzed. Prior data showed altered protein metabolism after 30 min, so in this study we evaluated 30 min with more extensive hypobaric hypoxia as well as a doubled time period (60 min).

### 3.1. 30HH vs. 30GC

The proteins GC, PLG and CFHR1 were found significantly expressed differently. Each protein was also found altered in the 60 min comparison. Vitamin D-binding protein belongs to the albumin gene family, and it transports vitamin D metabolites between skin, liver and kidney and then to the various target tissues. As a Gc protein-derived macrophage activating factor, it is a Macrophage Activating Factor (MAF) that has been tested for use as a cancer treatment that would activate macrophages against cancer cells [15]. Other authors found equal alteration for GC [1]. The plasminogen protein (PLG) encoded by this gene is a serine protease that circulates in blood plasma as an inactive zymogen and is converted to the active protease, plasmin, by several plasminogen activators such as tissue plasminogen activator (tPA), urokinase plasminogen activator (uPA), kallikrein and factor XII (Hageman factor). The Complement-factor-H-related-protein 1 (CFHR1) showed to be upregulated in the 30HH. CFHR1 is a complement regulator which has been reported to regulate complement by blocking C5 convertase activity and interfering with C5b surface association. Bio-informatical network analysis using GeneMania did not identify a significantly altered pathway.

### 3.2. 60HH vs. 60GC

A total of 18 differently expressed protein spots were identified by the 2D DIGE-Gel, concluding nine different proteins (ALB; CFI; C3; CFHR1; C1QB; GC; HPX; PLG; SERPINF1). ALB (n = 8) and CFHR1 (n = 2) were expressed differently in more than one protein modification, resulting in post-translational modification. The three proteins from HH30 (GC; PLG; CFHR1) were also altered during HH60, but with different expressive behavior. For example, PLG, downregulated in 30HH, showed to be upregulated in HH60. Hemopexin (HPX) was significantly upregulated in HH60. Hemopexin is the plasma protein with the highest binding affinity for heme [16]. The Complement factor I (CFI), also known as C3b/C4b inactivator, was significantly upregulated after HH60 in comparison to GC60. Complement factor I is a protein that, in humans, is encoded by the CFI gene. Complement factor I (factor I) is a protein of the complement system, first isolated in 1966 in guinea pig serum, that regulates complement activation by cleaving cell-bound or fluid phase C3b and C4b [17]. Pigment epithelium-derived factor (SERPINF1) was upregulated after 60HH. It is a neurotrophic protein that induces extensive neuronal differentiation in retinoblastoma cells and is also a potent inhibitor of angiogenesis [18]. Furthermore, complement C1q sub-component sub-unit B-protein (C1QB) was upregulated. This gene encodes the B-chain polypeptide of the serum complement sub-component C1q, which associates with C1r and C1s to yield the first component of the serum complement system. C1q deficiency is associated with lupus erythematosus and glomerulonephritis [19]. Complement component C3 (C3), which has a central function in the complement system, was upregulated after HH60. C3 plays a central role in the activation of the complement system. Its activation is required for both classical and alternative complement activation pathways. People with C3 deficiency are susceptible to bacterial infection [20] and show affected fracture healing [21]. Albumin (ALB) was expressed differently after HH60. Seven modifications were upregulated; one was expressed higher in GC60. Human serum albumin is the main protein of human blood plasma. It makes up around 50% of human plasma proteins. It binds water, cations (such as Ca^2+^, Na^+^ and K^+^), fatty acids, hormones, bilirubin, thyroxine (T4) and pharmaceuticals, including anesthetics. Its main function is to regulate the oncotic pressure of blood [22].

Given that the HIF pathway regulates the immune and inflammatory responses, the finding of this study may contribute to the altered immune cell activity through HIF-related pathways affected by exposure to hypobaric hypoxia [23]. The connection between hypoxia and inflammation has become evident, as HIF also confers responses to immune stresses [24]. Many of the hypoxic adaptations could be shown to be driven by HIF-1α, which is a central regulator of the hypoxic response [25]. Hypoxia response elements containing HIF-1α binding sites were identified in genes encoding transferrin [26], vascular endothelium growth factor [27], inducible nitric oxide synthase and glucose transporter 1 (GLUT 1) [28]. All these are playing important roles in systemic, tissues or intracellular O_2_ homeostasis, allowing for increased anaerobic ATP synthesis [5].

Another hallmark of cellular responses to hypoxia is the upregulation of oxygen-independent metabolic pathways to supply the additional energy necessary for cell survival under diminished oxygen availability [29].

Previous studies demonstrated that in participants who were exposed to moderate hypoxia simulation of 2400 m or 8000 ft for 30 min in a hypobaric pressure chamber, protein expression of 14 spots was significantly altered [6]. Bio-informatic analysis revealed an association of the altered proteins with the signaling cascades “regulation of hemostasis” (four proteins), “metabolism” (five proteins) and “leukocyte mediated immune response” (five proteins). Even though hypobaric hypoxia was short and moderate, analysis of protein expression revealed an association to immune response, protein metabolism, and haemostasias [6]. One protein (C1QB), which was altered after 60 min of HH in this study, was already altered significantly after 30 min of exposure [6]. Thus, mild hypobaric hypoxia (8000 ft) affected the expression of proteins to a greater extent than 30 min of more severe hypobaric hypoxia (15,000 ft) for the equal period of exposure time.

Data of Wang et al. [30] from 2018 revealed that acute phase proteins and inflammatory cytokines (IL-1β, IL-6 and TNF-α) show significant changes if exposed to altitude conditions. A total of 104 male soldiers rapidly ascending from Beijing (20–60 m) to Germu, Qinghai (3200 m), were divided into an AMS (Acute-Mountain-Sickness) group and a non-AMS group according to the Lake Louis Score system. Forty-nine blood samples were collected before and on the third day after ascending to high altitude. Serum haptoglobin (Hp), transferrin (Tf) and complement C3 were detected by immune scattered nephelometry, whereas serum interleukin-1beta (IL-1β), IL-6 and tumor necrosis factor-α (TNF-α) were detected by chemical luminescence immunity analyzer. The combination of inflammatory cytokines or acute phase proteins improves the specificity for the diagnosis of AMS. This might provide objective indexes for scanning and screening individuals susceptible to AMS in the early stage of rapid ascending. Differences in physical indexes between the AMS group and the non-AMS group were of no statistical significance. In the AMS group, serum Tf significantly increased while Hp decreased when compared with the non-AMS group. Serum IL-1β, IL-6 and TNF-α were higher in the AMS group than in the non-AMS group.

By comparing the plasma proteins of high-altitude natives with those of a near sea level control group, Ahmad et al. [2] found several proteins with a significant alteration. The upregulated proteins were identified as vitamin D-binding protein, hemopexin, alpha-1–antitrypsin, haptoglobin β-chain, apolipoprotein A1, transthyretin and hemoglobin beta chain. The downregulated proteins were transferrin, complement C3, serum amyloid, complement component 4A and plasma retinol-binding protein. Since all the up- and downregulated proteins identified above are well-known inflammation inhibitors and play a positive anti-inflammatory role, these results show the presence of some adaptive mechanism that sustains the inflammation balance in high-altitude natives exposed to hypobaric hypoxia.

Pavlicek et al. [31] demonstrated that C3 and alpha1AT levels increased during hypobaric hypoxia (from 0.94 (0.11) g/L to 1.07 (0.13) g/L, and from 1.16 (0.08) g/L to 1.49 (0.27) g/L, respectively; *p* < 0.05) in concordance with results in this study.

### 3.3. Limitations

A broader understanding of hypoxia-induced alterations in cellular or organ function could be better achieved from a combined knowledge derived from the concerted application of genomics and proteomic approaches. A prior study from 2016 revealed that even though hypobaric hypoxia was short and moderate (30 min at 2400 m; comparable to an airliner flight in the pressure cabin), analysis of serum protein expression in human subjects revealed an association to immune response and hemostasis [6]. According to these results, the current study used an equivalent methodological approach to combine the results of both studies. It was shown that even moderate hypobaric hypoxia seems to influence the human immune system. The findings of this study indicate that proteomic changes are more likely to be time-dependent than to be affected by the extent of the hypobaric hypoxia. Until now, there has been no study investigating the difference between the duration and extent of hypobaric hypoxia and the effects on the human proteome file.

## 4. Material and Method

### 4.1. Ethical Approval

The present study was approved by the ethical committee of the University of Cologne, Cologne, Germany (No. 18-045). The study registry number on clinicaltrials.gov, National Library of Medicine, 8600 Rockville Pike, Bethesda, MD 20894 is NCT03823677 (19 April 2021).

### 4.2. Participants

A total of N = 80 male subjects (181.2 ± 11.0 cm and 84 ± 15 kg, 26 ± 6 years old) participated in the trial and were randomly assigned into four different groups (GC30, GC60, HH30 and HH60). Subjects were screened for any acute or chronic illness prior to being assigned to any of the study groups. Participants who had any cardiovascular; pulmonary; neurological; or ear, nose or throat diseases were excluded in advance from this study. Furthermore, subjects who had any cold symptoms did not meet the inclusion criteria and were, therefore, excluded.

### 4.3. Study Groups

**Figure 15:**
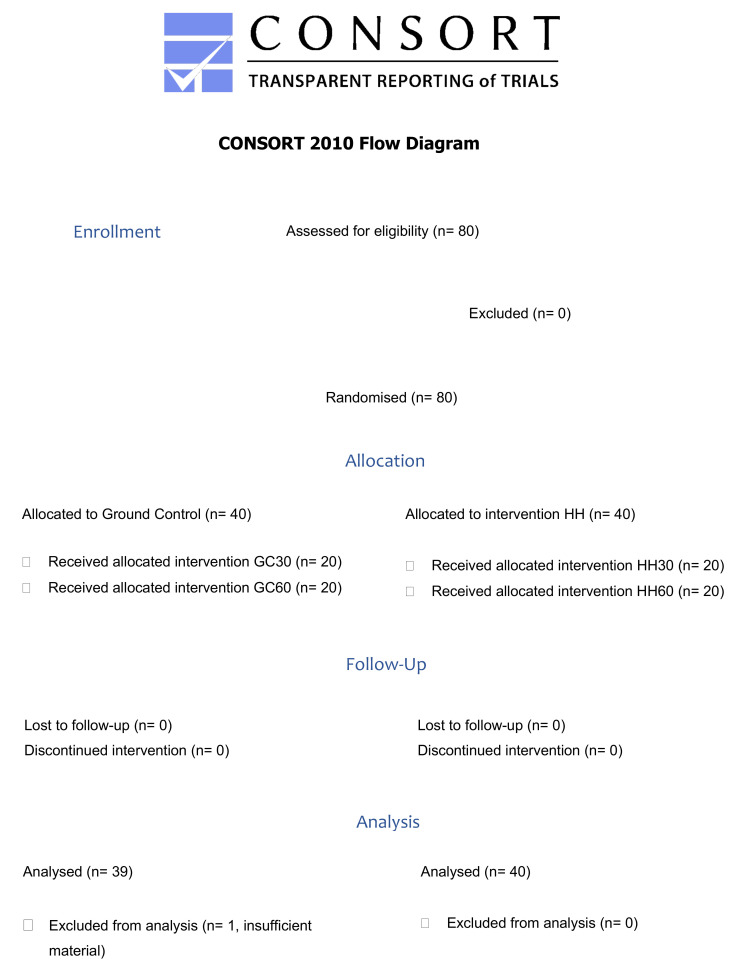
Consort 2010 Flow Diagram.

A total of N = 80 subjects were assigned randomly (by a lottery procedure with the trial participants randomly drawing study group abbreviations) to four different groups. A total of n = 40 subjects were assigned to the ground control (GC) group. Of these, n = 20 subjects were exposed 30 min in the pressure chamber without pressurization (GC30) and n = 20 subjects for 60 min in the pressure chamber without pressurization (GC60). A total of n = 40 subjects were assigned to the pressurized groups, where each batch of n = 20 subjects stayed in the pressurized pressure chamber for 30 min (G30) or 60 min (G60), respectively (Figure 15).

### 4.4. Experimental Design of the HH30 and HH60 Groups

**Figure 16:**
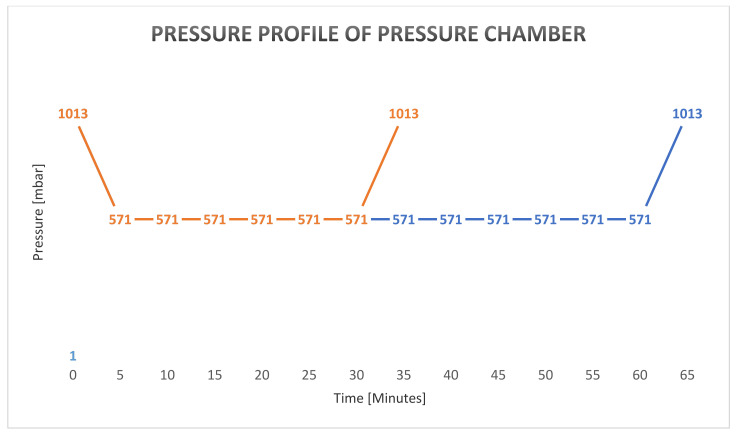
Pressure Profile of Pressure Chamber during Experimental Groups HH30 and HH60.

During the first 5 min after starting the pressure chamber (HAUX, Karlsbad, Germany, accessible for one person and operated by automatic pressure profile for each participant), surrounding pressure was reduced by 85.6 mbar/min until the inside pressure reached 571.82 mbar, equal to an altitude of 4572 m (15,000 ft) during the 30/60 min experiments. For the re-compression phase, the same gradient was applied (Figure 16).

### 4.5. Samples

After hypobaric hypoxia, serum was drawn and centrifuged at 5000× *g* for 5 min for further analysis. Serum samples were stored at −80 °C until proteomic analysis. All samples used in this study were prepared within 30 min of sample collection and showed no signs of hemolysis.

The intact arrival of the samples prior to analysis was confirmed by the laboratory. One blood sample was insufficient due to an inadequate amount of protein and was, therefore, excluded from the analysis.

### 4.6. Protein Analysis

Analysis of proteins was performed by an external, ISO-certified laboratory (TOPLAB GmbH, ISO 9001:2015, Martinsried, Germany).

### 4.7. Albumin and Immunoglobulin Depletion

To enrich for lower abundant proteins and therefore allow detection of differences in protein pattern of the serum samples high abundant serum, proteins were depleted using immunobeads. The use of a High SelectTM Top14 Abundant Protein Depletion Mini Spin Resin aimed to remove the following 14 proteins from human serum: human serum albumin (HSA), albumin, IgG, IgA, IgM, IgD, IgE, kappa and lambda light chains, alpha-1-acid glycoprotein, alpha-1-antitrypsin, alpha-2-macroglobulin, apolipoprotein A1, fibrinogen, haptoglobin and transferrin. Samples were thawed at RT, and per experimental group, 50 μL of each dedicated serum sample were pooled. From each pooled sample, 4 × 10 μL serum were applied to four High SelectTM Top14 Abundant Protein Depletion Mini Spin Columns (Pierce) and incubated for 10 min on a rotary wheel. The flow-through of the four columns per sample that contained the depleted serum was re-combined, and the success of the depletion was analyzed by SDS PAGE and BCA assay.

### 4.8. BCA Protein Assay

The protein concentration of samples was determined using a BCA Assay. A PierceTM BCA Protein Assay Kit (#23225) was used for this method. Two different dilutions of the depleted serum samples (1:10 and 1:20) were measured with the assay, which was performed in duplicates. Further, a BioSpectrometer^®^ Basic (Eppendorf, Hamburg Germany) was used as photometer, and standard curve and protein concentrations were calculated by the instrument software.

### 4.9. 2D SDS PAGE

First, 5 μL of each pooled serum samples prior to depletion were diluted with 250 μL of water (HPLC grade) and 245 μL 3× reducing SDS sample buffer (30% glycerol; 187.5 mM Tris pH 6.8; 6% SDS, 3% DTT). Then, 1/25 of the serum samples after depletion (~55–60 μL) was diluted with 30 μL 3× reducing SDS sample buffer (30% glycerol; 187.5 mM Tris pH 6.8; 6% SDS, 3% DTT). Samples were heated for 5 min at 90 °C.

Separation was performed on a pre-cast Tris-Glycine gel T(%) 4-20 from Serva (SERVAGelTM TG 4-20, cat. No. 43230.01). Per lane, 5 μL of the prepared samples prior depletion and 1/3 of the prepared samples after depletion (27–30 μL) were loaded onto the gel. To estimate the molecular weight of the proteins, 5 μL of a molecular weight marker commercially available from Serva (protein test mixture 6, cat. No. 39207.01) and 5 μL of a pre-stained protein marker (Serva Triple Color Protein Standard III, cat. No. 39258) were also loaded per gel. The un-stained Serva standard comprised masses corresponding to 97, 67, 45, 29, 21, 12.5 and 6.5 kDa, respectively. The pre-stained standard comprised blue-labeled proteins at 245, 180, 135, 100, 63, 48, 35, 20, 17, 11 and 5 kDa; a red-labeled protein at 75 kDa; and a green-labeled protein at 25 kDa. Lanes without protein load were filled with 1x sample buffer instead to assure an even gel run.

The gel run was started at low power for 20 min at 10 mA. The main separation took place at 25 mA with the current limited at 500 V. After 90 min, the dye front had reached the rear of the gel, and the gel run was stopped.

The gel was stained with colloidal Coomassie overnight (5% aluminum sulfate hydrate, 10% ethanol, 0.02% Coomassie G250, 2% o-phosphoric acid). After de-staining with de-ionized water until appropriate background reduction, the gel was scanned with a visual scanner (CanoScan9900F) using a resolution of 300 dpi.

### 4.10. 2D DIGE—Re-Buffering and De-Salting

The protein samples used for 2D PAGE had to be re-buffered and concentrated to remove interfering substances and to allow sufficient protein load. According to the protein determination results with BCA assay, 100 μg protein of each depleted serum sample was diluted with labeling buffer (30 mM Tris, pH 8.5; 7 M urea; 2 M thiourea, 4% CHAPS, Roche cOmplete protease inhibitor cocktail, Pefablock SC protease inhibitor) and re-buffered using Vivaspin 500 ultrafiltration devices with a cut-off of 5 kDa. After three times of dilution and volume reduction, the samples were present in 75 μL labeling buffer at last with a protein concentration of 1.33 mg/mL.

### 4.11. DIGE Labeling

Labeling of the proteins was performed using SERVA Lightning SciDye Kit (Serva cat. No. 43407.01) to covalently bind fluorescent dye to primary amino groups such as Lysine and protein N-terminus. The Serva dyes Sci2, Sci3 and Sci5 are equal to Cy2^®^, Cy3^®^ and Cy5^®^ that are trademarks of GE Healthcare Company. First, 50 μg of the samples Control I and Control II was labeled with Sci3, and 50 μg of the samples Hypoxia 30 min and Hypoxia 60 min was labeled with Sci5. Additionally, 25 μg of each sample was pooled as the internal standard sample (Pool-A) and divided into two aliquots with 50 μg pooled protein each. These 2 × 50 μg internal standard samples were each labeled with Sci2. For each labeling reaction with Sci2, Sci3 and Sci5, 50 μg protein (16.7 μL) was applied to 400 pmol SciDye. The reactions were performed on ice for 30 min and stopped by adding lysine and incubating for another 10 min. Labeling was performed according to the manufacturer’s protocol. The labeling chemistry is based on minimal labeling of lysine residues of the proteins, with one lysine residue labeled per protein on average.

After labeling, reactions to run in the same 2D DIGE gel were pooled. The final volume of each sample was adjusted to 140 μL with lysis buffer B (7 M urea; 2 M thiourea; 4% CHAPS; Roche cOmplete protease inhibitor cocktail, Pefablock SC protease inhibitor), and samples were supplemented with 2% Servalyte and 1% DTT. Finally, 180 μL of re-hydration buffer (6 M urea; 2 M thiourea; 2% zwitterionic surfactant; 1% dithiothreitol; 1 *v*/*v* % Servalyte 3-10 Iso-Dalt for 2D, Roche cOmplete protease inhibitor cocktail, Pefablock SC protease inhibitor) was added to each sample.

### 4.12. 2D Gel Electrophoresis

For the 2D DIGE experiment, samples were loaded directly after labeling onto two 18 cm IPG strips pH 3-10NL from Serva (T = 4%, C = 2.7%) using passive in-gel re-hydration for the sample application. Therefore, the IPG strips were re-hydrated with 320 μL sample for 16 h at room temperature. IEF was performed for ~83 kVh in total. All steps were limited to 75 μA per strip and performed at 20 °C. The applied instrument was an IEF 100 focusing unit from Hoefer. After focusing, the IPG-strips were treated for 10 min with a fresh solution of DTT for protein reduction. Subsequently, alkylation of free sulfhydryl groups was performed by exposing the strips to an iodoacetamide solution for another 10 min. The proteins were separated in the second dimension on hand-made gels (T = 13%, C = 2.6%) with an SDS-Glycine-Tris buffer as running buffer. The two 2D DIGE gels were run in parallel. A molecular weight standard (SERVA Electrophoresis GmbH, protein test mixture 6) was used as control of the second dimension. The standard was applied onto the gels adjacent to each IPG strip with masses corresponding to 97, 67, 45, 29, 21, 12.5 and 6.5 kDa, respectively.

### 4.13. Image Acquisition

To visualize the labeled and separated proteins after electrophoresis, the 2D DIGE gels were scanned at a resolution of 100 μm with a Typhoon FLA 9500 (GE Healthcare). The settings for the scans were as follows: Sci2: laser: 473 nm; Sci3: laser: 532 nm; Sci5: laser: 635 nm filter (nm): 530 DF20 filter (nm): 570 DF20 filter (nm): 665 LP.

Subsequently, to visualize the total protein load, 2D DIGE gels were stained with colloidal Coomassie overnight (5% aluminum sulfate hydrate, 10% ethanol, 0.02% Coomassie G250, 2% o-phosphoric acid). After de-staining with de-ionized water until appropriate background reduction, gels were scanned with a visual scanner (CanoScan9900F) using a resolution of 300 dpi.

### 4.14. Image Analysis

For image analysis, scan files of the 2D-DIGE gels were loaded into Delta 2D image analysis software (Decodon, Version: 4.8).

For the image analysis, the workflow given by the software was followed. First, the images were grouped into four groups according to sample load. Each group contained only one gel: Control I in the first group (orange); hypobaric hypoxia 30 min in the second group (red); Control II in the third group (turquoise) and hypobaric hypoxia 60 min in the fourth group (blue). The images of the internal standard were grouped separately (yellow).

Next, the images were warped to each other. This action is performed prior to spot detection and is used to eliminate distortions between the gels. The warp strategy “In-Gel Standard Warping” was used. Specifically, the Cy2 gel of every sample was warped with the Cy3 and Cy5 images of the same gel. Additionally, the Cy2 images of each gel were warped with each other (see Figure 3). The warping was performed automatically but inspected and approved manually. Subsequently, a fusion image created from all Cy3 and Cy5 images was generated. The spot information present in the fusion image is based on the average spot intensities from all images. Spot detection was performed exclusively in the fusion image since it contained all spots present in the experiment. The detection parameters were adjusted to minimize false positive detection of dust and dye speckles and to yield a total spot count of 500–800 spots. The result of the automatic spot detection was inspected and manually edited. Only minor changes to the spot detection had to be made. The finally detected spot pattern of the fusion image was then transferred to all other images in the experiment. As a result, gels contain the same spot number and pattern.

A quantitative comparison of the spot intensities was performed between the gel images of the hypobaric hypoxia treated samples and the corresponding controls. The ratio of spot volumes was calculated for each spot of the comparison. In case a value of 2.0 was calculated for the ratio, this means the spot intensity did increase two times compared to the reference, a ratio of 0.5 means a decrease of 2-fold. Unchanged spot intensities have ideally a ratio of 1.0. The ratio of 2-fold was set as a threshold for differences of the sample material and above technical variability. Using filter settings for this threshold, quantitative differences in the spot intensities between two samples were identified.

### 4.15. Bio-Informatic Analysis of Proteins

As a first step, statistically significantly regulated proteins from blood were identified and analyzed by network analyses (GeneMania). Afterward, those statistically significant proteins were grouped (GC30, GC60, HH30 and HH60) using hierarchical cluster analysis (Perseus@). As a third step, proteins of similarly early upregulated clusters underwent further network analysis to evaluate possible corresponding proteins or functions in the blood. This approach to deal with pooled proteomic data is described in detail below.

### 4.16. Network Analysis of Proteins (GeneMania)

Proteins from a serum analysis which were significantly altered were used for further bio-informatical analysis to identify underlying networks, signaling cascades and pathways affected.

Biological functions of statistically significantly regulated proteins were identified using functional network analysis. GeneMania (http://www.genemania.org, accessed on 26 June 2021) is a tool that helps predict interactions and functions of genes in terms of network and, when available, of pathways. It provides the possibility to customize the network and allows for the choosing of data sources or highlighting of specific functions with a more comfortable graphic experience. It is developed and continually updated by the University of Toronto and is funded by the Ontario Ministry of Research and Innovation [13]. GeneMania knowledge is based on data from large databases, which comprise Gene Expression Omnibus, BioGRID, EMBL-EBI, Pfam, Ensembl, Mouse Genome Informatics, the National Center for Biotechnology Information, InParanoid and Pathway Commons [14]. It was developed for making predictions about a gene or protein function based on a query of a list of proteins that share a function of interest. The software allows for taking advantage of the persistent improvement and proliferation of high-throughput genomics and proteomics datasets by making up-to-date predictions of their interaction with other genes or proteins [13,14].

As these software programs use different algorithms, we decided to perform the bio-informatical analyses with all of them in order to retrieve the highest number of predicted interactions, maintaining an acceptable level of confidence (0.400).

The associated functions detected by the software were downloaded in TAB-separated-values format and exported to Microsoft Excel (Microsoft, Redmond, WA, USA; version 2007), where they were filtered into sub-groups, which were re-analyzed using GeneMania.

## 5. Conclusions

In comparison to HH30, HH60 had distinct differences in the number of differential protein spots (noticeably more proteins due to the longer time of hypoxia). There are indicators that a change in proteins is dependent on time but not necessarily on the extent of the hypobaric hypoxia, as mild hypobaric hypoxia (8000 ft) affected the expression of proteins to a greater extent than 30 min of more severe hypobaric hypoxia (15,000 ft) for the same duration in this study. The mechanisms of alterations in hemostasis and immune system due to staying at a high altitude can also be seen in the levels of protein metabolism. Results of the present study may give input for developing molecular tests for the identification of hypoxia-induced problems during sustained higher altitudes.
